# Effects of different harvesting times and processing methods on the quality of cultivated *Fritillaria cirrhosa* D. Don

**DOI:** 10.1002/fsn3.2241

**Published:** 2021-03-15

**Authors:** Bujin Ma, Jing Ma, Bo Li, Qian Tao, Jiaxia Gan, Zhuyun Yan

**Affiliations:** ^1^ State Key Laboratory of Characteristic Chinese Medicine Resources in Southwest China School of Pharmacy Chengdu University of Traditional Chinese Medicine Chengdu China

**Keywords:** alkaloid, *Fritillaria cirrhosa* D. Don, Fritillariae Cirrhosae Bulbus (FCB), harvest period, postharvest processing methods

## Abstract

*Fritillaria cirrhosa* D. Don is the major source plants of traditional Chinese medicine Fritillariae Cirrhosae Bulbus (FCB). Domestication, introduction, and cultivation is an important strategy to alleviate the shortage of endangered medicinal plants of *F. cirrhosa*. However, until now, the yield and quality changes of FCB in different harvest periods and drying treatments after harvest were not well understood. Therefore, in this paper, we investigated the yield and quality of cultivated *F. cirrhosa* at different harvest periods and postharvest processing methods. The results showed that dry weight per bulb ranged from 0.8913 to 1.4681 g and reached the highest at the wilting stage. The soluble sugar content ranged from 0.075% to 0.127% and reached the highest at the wilting stage. The content of total alkaloids ranged from 0.088% to 0.218% and reached the highest at the late‐flowering stage. The contents of peimisine, sipeimine, peimine, and peiminine were 0.01178%‐0.02615%, 0%–0.01713%, 0%–0.00745%, and 0%–0.00621% and reached the highest at the late‐flowering period, wilting period, young fruit period, and initial flowering period, respectively. For the two different postharvest processing methods, the contents of total alkaloids and the 16 main characteristic peaks did not exhibit significant differences. Still, the alkaloid contents of the oven drying after washing were slightly higher than the sun drying. In conclusion, the best harvest period is the wilting period of *F. cirrhosa*, and oven drying after washing is more beneficial to ensure the quality of FCB and improve productivity.

## Graphical abstract

Different harvest periods had a greater impact on the bulb yield and quality of *F. cirrhosa*, while the alkaloid ingredients in the bulbs were not affected by the processing method and processing time after harvest.

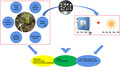

## 1 INTRODUCTION

The genus of *Fritillaria* (Liliaceae) contains about 140 species of perennial herbs, and about 80 species are distributed in China (Chen et al., 2019; Fan et al., 2019). Most of the dry bulbs of *Fritillaria* species are commonly used in traditional Chinese medicine (TCM) for the treatment and prevention of respiratory diseases. The earliest record could be traced back to the medical classics of *Shennong Bencao Jing* (*The Divine Farmer′s Materia Medica Classic*) in the 1st century AD (Guo et al., 2017). According to the different origins and treatment symptoms, the Pharmacopoeia of the People′s Republic of China includes five types of *Fritillaria* medicinal materials: Fritillariae Cirrhosae Bulbus (FCB), Fritillariae Thunbergii Bulbus (FTB), Fritillariae Ussuriensis Bulbus (FUB), Fritillariae Pallidiflorae Bulbus (FPB), and Fritilariae Hupehensis Bulbus (FHB). Among them, the original plants of the FCB mainly come from the dry bulbs of six plants, including *F. cirrhosa* D. Don, *F. unibracteata* Hsiao et K. C. Hsia, *F. przewalskii* Maxim, *F. delavayi* Franch., *F. taipaiensis* P. Y. Li, and *F. unibracteata* Hsiao et K. C. Hsia var. *Wabuensis* (S. Y. Tang et S. C. Yue) Z. D. Liu, S. Wang et S. C. Chen (State Pharmacopoeia Committee., 2020a). As a critical FCB source, *F. cirrhosa* has always been regarded as a representative of good quality and high production added value (Wang et al., 2016b).

FCB mainly contains steroidal alkaloids, nucleosides, saponins, terpenoids, and many other compounds (Duan et al., 2011; Wang et al., 2017). Several studies have shown that alkaloids in *F. cirrhosa* extracts are the main active ingredients, which have the effects of antitussive, expectorant, and antiasthmatic (Guo et al., 2020; Wang et al., 2016a). In recent years, studies have found that they also appeared to have antitumor, anti‐inflammatory, and antihypertensive pharmacological effects, which have further broadened its application scope (Guo, Wu, et al., 2020; Li et al., 2020; Zhao et al., 2018). However, the natural reproductive capacity of the wild *F. cirrhosa* is low, and the population self‐renewal is slow. Coupled with uncontrolled harvesting in recent years, there has been a situation of short supply, causing its price to soar, reaching about 3,100 CNY (= 449 US$) per kg in 2020 (Tiandi Network of Traditional Chinese Medicine., 2020). Nowadays, artificial cultivation has become an effective way to supplement the wild resources of *F. cirrhosa* to meet clinical medicine needs (Cunningham et al., 2018). However, until now, we still know little about the ingredient content of the cultivated *F. cirrhosa*.

Moreover, the cultivation of *F. cirrhosa* could not only effectively alleviate the resource pressure of wild plants but also could improve the yield of medicinal materials and the content of active substances by changing plant growth conditions and standardizing postharvest processing methods. Among them, the appropriate harvest period is regarded as one of the essential factors to affect the yield and quality of medicinal material during production (Alqahtani et al., 2015). Some studies were employed to investigate the content changes of active ingredients during the growth of *F. cirrhosa* in the wild or the tending condition (Konchar et al., 2011; Liu et al., 2009). However, there was no report about the ingredient changes in different phenological periods under artificial cultivation conditions until now. Besides, postharvest processing also plays an essential role in the production of herbal medicines and may affect the organoleptic and chemical properties, as well as clinical efficacy and safety (Zhu et al., 2018). It has been reported that there are more than five postharvest processing methods of *F. cirrhosa* bulbs, among which sun drying and low‐temperature drying are the two main methods that are easy to operate and labor‐saving in practice (Huang et al., 2009; Li et al., 2018). Therefore, investigating the effects of different postprocessing methods and drying times on the change of active ingredients in bulbs could remarkably promote the production of *F. cirrhosa* and alleviate the shortage of *F. cirrhosa* supplement.

In this study, we have systematically investigated the content changes of active ingredients in different harvest periods and postharvest processing methods, and analyzed the most suitable harvest period and postharvest processing method. Our study will provide detailed information for quality control of artificial cultivated *F. cirrhosa*.

## 2 MATERIALS AND METHODS

### 2.1 Plant material and growth conditions

The research site was located in the *Fritillaria* planting base of Qinghai Lvkang Biotechnology Development Co., Ltd. (36°59′E, 101°59′N, the altitude of 3,050 m), which belongs to the alpine meadow area of the Qinghai‐Tibet Plateau. Three‐year‐old *F. cirrhosa* bulbs as the breeding source were cultivated by Qinghai Lvkang Biological Development Co., Ltd. The experimental plot border width was set at 1 m, trench depth 12 cm, row spacing 15 cm, and cultivation density 60 plants/m^2^. All the materials were managed normally.

### 2.2 Harvest and processing methods

The entire growth cycle of *F. cirrhosa* included the germination period (H1), initial flowering period (H2), late‐flowering period (H3), young fruit period (H4), fruit maturity period (H5), and wilting period (H6). Ten bulbs were harvested randomly in each growth stage, immediately placed in liquid nitrogen, and then stored at −80°C (Table 1). The dried samples were milled into powder, sieved through 80 mesh before used for content determination.

**TABLE 1:** Changes in individual dry weight, water content, soluble sugar content, and total alkaloid content in bulbs of cultivated *F. cirrhosa* at different phenological stages

Sample No.	Phenological stages	Individual dry weight (g) *n* = 10	Water(%) *n* = 3	Soluble sugar^a^, ^b^ (%) *n* = 3	Total alkaloid^a^ ^,^ ^c^ (%) *n* = 3
H1	Germination period	0.9364	75.52	0.115 ± 0.006b	0.088 ± 0.004e
H2	Initial flowering period	0.8913	71.27	0.092 ± 0.004c	0.201 ± 0.006b
H3	Late‐flowering period	0.9762	70.46	0.075 ± 0.012d	0.218 ± 0.002a
H4	Young fruit period	1.2221	66.73	0.092 ± 0.006c	0.125 ± 0.009d
H5	Fruit maturity period	1.3652	65.45	0.112 ± 0.006b	0.135 ± 0.006 cd
H6	Wilting period	1.4681	72.25	0.127 ± 0.006a	0.140 ± 0.008c

^a^ Results are presented as mean of three replicates ± standard deviation. Different letters within each column indicate means significantly different according to Duncan's test (*p* <.05).

^b^ Results are expressed as glucose equivalents (g of glucose/g of sample in dry weight).

^C^ Results are expressed as sipeimine equivalents (g of sipeimine/g of sample in dry weight).

Materials in wilting period (H6) were used for postharvest processing studies. Briefly, (1) Sun drying: about 300 g of fresh bulbs was put into a container and dried under sunlight for 15 days. Samples of 40 g (P1‐P5) were collected every 3 days and stored at −80℃ for further research. (2) Oven drying after washing: the bulbs were washed with running water to remove the soil, and the materials were placed in an oven at 60℃ after the water droplets drained away. Samples of 40 g (P6‐P9) were collected every 6 hr and stored at −80℃ for further research (Table 3). Samples of 20 g treated by different drying treatments were placed in a grinding miller (IKA, Germany) grounded to powder with liquid nitrogen before being used for moisture and alkaloid analysis.

### 2.3 Determination of moisture content

The moisture content of the samples was detected by reference to the method of Chinese Pharmacopoeia (State Pharmacopoeia Committee., 2020b). Approximately 2.0 g of sample powder was dried in ovens (Langgan, China) at 105℃ for 5 hr and then weighed. The drying and weighing were continued until the difference between two consecutive weightings was within 0.005 g, and the measurement was repeated three times for each sample.

### 2.4 Determination of soluble sugar content

Soluble sugar was extracted and quantified according to the method described by literature (Ma et al., 2009). About 50 mg of sample powder was extracted with 10 ml of 80% ethanol in a water bath (70°C) for 30 min. After cooling to room temperature and centrifuged (Cence, China), the operation was repeated once and the supernatants were combined. The reaction mixture contained 2 ml of diluted supernatants, 1 ml of 2% anthrone reagent, and 10 ml concentrated H_2_SO_4_ and was heated in a water bath (100°C) for 1 min. After the reaction, the absorbance of each sample was measured at a wavelength of 620 nm using an ultraviolet spectrophotometer (Aoe, China). The soluble sugar content was measured using a calibration curve, which was constructed by using the glucose solution. Data were expressed as gram of glucose equivalents for per gram dry weight (DW).

### 2.5 Determination of total alkaloid content

The method recorded in Chinese Pharmacopoeia was used to determine the total alkaloids of *F. cirrhosa* bulbs (State Pharmacopoeia Committee., 2020a). Dried sample powder of 2 g was accurately weighed and placed in a 50 ml conical flask with stopper, then added 2 ml of ammonia solution and standing for 1 hr. Subsequently, 40 ml of chloroform‐methanol (4:1, V/V) mixture solution was added, and the extract was refluxed at 80°C in a water bath with heat for 2 hr. The extract was vacuum suction filtered and evaporated, then dissolved and diluted with methanol to a 50 ml volumetric flask. Finally, the determination of total alkaloids was based on the reaction between alkaloid and bromocresol green (BCG), and after the reaction was completed, the absorbance was determined at 415 nm (Aoe, China). The standard curve was plotted with sipeimine solution, and the total alkaloid content of each sample was calculated. Data were expressed as gram of sipeimine equivalents for per gram DW.

### 2.6 Determination of alkaloids by HPLC‐ELSD

#### 2.6.1 Preparation of standard solution

The 5.0 mg of each alkaloid reference substance, peimisine, sipeimine, peimine, and peiminine, was accurately weighed into a 5 ml volumetric flask and made up to the volume with methanol. Different volumes (1, 2, 5, 10, 15, and 20 μl) of mixed standards solution were taken into the HPLC system to measure the peak areas. The linear regression equation of each reference substance was obtained by taking the logarithm of the peak area of mixed standards solution and the injection volume. The mixed solutions were filtered through a 0.45 μm microporous membrane before injection into the HPLC system.

#### 2.6.2 Preparation of sample solution

The samples were prepared for alkaloid analysis as described by Wang (2013) with minor modifications. An accurately weighed sample of 2.0 g dried powder and 4 ml of ammonia solution were added in a 100 ml round‐bottomed flask for 2 hr. Then, the extraction and concentration operations were the same as 2.5. Finally, the extract was diluted to a 2 ml volumetric flask with methanol. The samples were filtered through a 0.45 μm Millipore filter membrane before injection.

#### 2.6.3 Chromatography conditions

The HPLC‐ELSD conditions were used for the determination of the alkaloids, and an LC‐20AT Shimadzu high‐performance liquid chromatography system (Kyoto, Japan) equipped with an Alltech 2000 evaporative light‐scattering detector (Grace,) was used. The chromatographic separations were performed over a ZORBAX Extend‐C18 (250 mm × 4.6 mm, 5 µm; Agilent) column at a column temperature of 25℃. The column was eluted with a mixture of acetonitrile (mobile phase A) and water‐ammonium bicarbonate (PH = 10.10, mobile phase B) at a flow rate of 1.0 ml/min. The elution conditions were as follows: 0–10 min, 75%A to 60% A; 10–20 min, 60% A; 20–30 min, 60% A to 30% A; 30–45 min, 30% A to 10% A; 45–55 min, 10% A; 55–60 min, 10% A to 75% A; 60–65 min, 75% A. The drift tube temperature of the ELSD was set at 115℃, and using nitrogen as the carrier gas at a flow rate set at 3.0 L/min, the gain value was 2, and the injection volume was 20 µl for analysis. The concentration of each compound was calculated based on external standard method, using the calibration curves of each compound.

### 2.7 Statistical analysis

All experiments were performed at least three times, and data were analyzed using IBM SPSS 26.0 (IBM Corp) and Microsoft Excel 2010 (Microsoft Corp). The variance was analyzed by one‐way ANOVA, followed by Duncan's test for multiple comparisons. *p* <.05 was considered significant.

## 3 RESULTS AND DISCUSSION

### 3.1 Different harvest periods

#### 3.1.1 Biomass

At each harvesting period, ten bulbs were used to determine the fresh weight and dry weight, and the water content was calculated (Table 1). The results showed that the bulbs' dry weight decreased to a minimum value of 0.8913 g from germination to initial flowering. Still, from late flowering to the wilting stage, the dry weight of bulbs showed an increasing trend, reaching a maximum of 1.4681 g.

#### 3.1.2 Soluble sugar content

Soluble sugars (glucose, sucrose, and fructose) play an important role in the life cycle of plants. They could not only provide energy and mid‐metabolites but also act as signals to regulate plant growth and gene expression (Sami et al., 2016; Wang & Tang, 2014). So, we detected the soluble sugar content of *F. cirrhosa* bulbs at the different harvest stages. As shown in Table 1, the soluble sugar contents in *F. cirrhosa* bulbs gradually decreased from the germination stage to the late flowering stage and then steadily increased, reaching the highest at the wilting stage.

As we all know, sugars are largely produced in functional green leaves via photosynthesis, and most harvested organs, including seeds, fruits, tubers, corm, and bulbs, depending on an external supply of sugars for growth and development (Liu et al., 2020). We speculated that fewer carbohydrates were produced by photosynthesis from the germination period to the end of the flowering period. The aboveground part growth and development partly benefit from the nutrients (nonstructural carbohydrates) provided by the underground bulb (Martínez‐Vilalta et al., 2016). After pollination and fertilization of the plant, the stems and leaves no longer grow. Subsequently, the photosynthesis products are only used for fruit growth, and the excess sugars are transported by the sieve tubes to the underground part for bulb growth and storage (Braun et al., 2014). So, when the fruit ripens, the soluble sugar content in bulbs gradually rises to a level close to the germination period, making final preparation for the subsequent long‐term overwintering.

#### 3.1.3 Total alkaloid contents

Total alkaloid is an essential index in the quality control of BFC. In this study, the total alkaloid contents in different harvest periods were analyzed. The results showed that the total alkaloid contents in the bulbs of *F. cirrhosa* increased rapidly from the germination stage (0.088%), reached the highest level around the late flowering period (0.218%), and then decreased rapidly from the late flowering stage to the young fruit stage (0.125%). However, the total alkaloid contents increased again from the fruit maturity stage (0.135%) to the wilting stage (0.140%). It was evident that total alkaloid contents were significantly higher in flowering periods than in other growth periods. The same results were also found in the study of two Turkish *Hypericum* species (Cirak et al., 2013), *Origanum vulgare* L. (Towler & Weathers, 2015), and *Artemisia annua* L. (Baranauskiene et al., 2013). Through their study, it was found that the main active ingredients contained therein were all highest during the reproductive phase (around the flowering period). The reason may be that the reproductive activity of plants attracted insects, and their contact with plants stimulated the production of compounds, which was thought to be part of the plant's defense mechanism (Ribeiro et al., 2019).

According to the research of Konchar et al., (2011), the reproductive stage of the plant had a significant impact on the content of alkaloids. The content of the two main active alkaloids from wild *F. cirrhosa* plants was the highest at the fruit development stage and decreased significantly as the fruit matures. A study by Liu et al., (2009) showed that the total alkaloid contents reached the maximum when the fruit was mature, which was a reasonable harvest period for *F. cirrhosa* in the wild tending state. These differences might be attributed to the different growing conditions (environmental and developmental factors), developmental stage at harvest (age), postharvest treatment, storage conditions as well as the extraction and quantification methods used for bulbs of *F. cirrhosa*.

#### 3.1.4 Content of four monomeric alkaloids

Since each medicinal product has its content requirement for different bioactive compounds, the best harvest time might be identified according to the accumulation dynamics of target compounds (He et al., 2010). Isosteroidal alkaloids as target compounds are usually regarded as chemical markers in FCB or related medicinal products for quality control (State Pharmacopoeia Committee., 2020a; Wu et al., 2018). To investigate the dynamic changes of different alkaloid monomers in the various harvest periods, we have detected the contents of them, respectively. The results showed that the changing trend of the four alkaloid monomers was basically the same as the total alkaloid content except for sipeimine, whose peak value was in the wilting period. The other three alkaloid monomer contents had their maximum values around the flowering period of the plants (Table 2). This result was further evidence that the production or translocation of defensive compounds (alkaloids in this study) was not only inducible by stressful situations but also was related to specific plant reproduction stages, such as the beginning of flowering or fruit development.

**TABLE 2:** Changes in the content of four alkaloid monomers in bulbs of cultivated *F. cirrhosa* at different phenological stages

Sample No.	Peimisine (%, g/g DW)	RSD (%)	Sipeimine (%, g/g DW)	RSD (%)	Peimine (%, g/g DW)	RSD (%)	Peiminine (%, g/g DW)	RSD (%)
H1	0.01274	0.67	0.00693	1.47	0.00512	1.29	‐	‐
H2	0.02453	0.81	0.00315	1.62	0.00426	0.72	0.00621	1.17
H3	0.02615	0.95	0.00962	1.09	0.00564	1.11	0.00515	1.08
H4	0.01526	0.92	0.00694	2.31	0.00745	1.78	0.00324	0.92
H5	0.01178	0.68	‐	‐	‐	‐	‐	‐
H6	0.01351	1.01	0.01713	1.55	0.00474	2.63	0.00322	2.18

Note:

“–” means not determined.

Based on our data, we suggested that the most appropriate period to harvest the cultivated *F. cirrhosa* was the wilting period. At this time, the amount of dry matter can reach the maximum, the active substance content can be maintained at a high level, and the seeds can be harvested to expand the production scale.

### 3.2 Different postharvest processing methods

#### 3.2.1 Water loss

The changes in sample water loss under different postharvest treatments were investigated (Table 3). For the sun drying method, the results showed that the moisture content could meet the requirements of Chinese Pharmacopeia through 15 days of treatment (12.58% < 15%). By contrast, after 18 hr, the moisture content of oven‐dried samples could satisfy the pharmacopeia standard (13.96% < 15%).

**TABLE 3:** Changes of water content and total alkaloid content in bulbs of cultivated *F. cirrhosa* during drying treatment

Sample No.	Treatments	Water(%)	Total alkaloid^a^, ^b^(%)
H6	Untreated	72.25	0.129 ± 0.014a
P1	Sun−3d	68.72	0.130 ± 0.019a
P2	Sun−6d	57.56	0.126 ± 0.016a
P3	Sun−9d	34.14	0.129 ± 0.022a
P4	Sun−12d	16.99	0.125 ± 0.012a
P5	Sun−15d	12.58	0.129 ± 0.007a
P6	Oven−6h	34.29	0.129 ± 0.017a
P7	Oven−12h	26.52	0.128 ± 0.019a
P8	Oven−18h	13.96	0.125 ± 0.017a
P9	Oven−24h	9.25	0.131 ± 0.007a

^a^ Results are presented as mean of three replicates ± standard deviation. Different letters within each column indicate means significantly different according to Duncan's test (*p* <.05).

^b^ Results are expressed as sipeimine equivalents (g of sipeimine/g of sample in dry weight).

#### 3.2.2 Total alkaloid contents

Due to the fact that certain active components of traditional Chinese medicine will be changed because of the influence of temperature, enzymes, residual moisture contents, or other factors during the drying or other processing processes (Tetteh et al., 2019; Zhu et al., 2018). Shi et al., (2017) reported the active and nutritional ingredients content in harvested medicinal chrysanthemum (*Chrysanthemum morifolium Ramat*) was not only related to drying methods but also significantly influenced by the specific conditions of each processing method. To investigate whether the total alkaloids of *F. cirrhosa* were changed under the different postharvest processing, we have compared the contents of total alkaloids in the two processing methods. Our results showed that the total alkaloids did not demonstrate a significant difference (*p* > .05) in the two postharvest processing methods as well as different processing times (Table 3).

It is widely known that drying will cause fluctuations in the content of active ingredients in plants. A lot of research has shown that secondary metabolites such as phenolics, flavonoids, tannin, terpenoids, and saponin were remarkably influenced by sun drying or oven drying processed (Ademiluyi et al., 2018; Ni et al., 2013). Meanwhile, the effect of drying on alkaloids also has been proved. Chen et al., (2016) investigated different drying methods (i.e., sun drying, sulfur fumigation drying, microwave drying, infrared drying, hot air drying, and freeze drying) on *Fritillaria thunbergii* Miq, and the results showed that the total content of peimine and peiminine was preserved to the maximum on hot air drying at 60℃. Huang et al., (2009) evaluated different postharvest processing methods on wild tending *F. cirrhosa*, and they observed the highest alkaloids (peimisine, peimine, and peiminine) content in the oven drying than other drying methods. These findings demonstrate the unique advantages of the oven‐drying method for bulb processing of genus *Fritillaria*. However, in our present study, whether it is sun‐dried or oven‐dried, or different drying time under the same drying method, it has little effect on the content of total alkaloids. The reason may be that alkaloids being stable compounds were unlikely to be degraded by ambient light and temperature under the relatively mild conditions of the current two processing methods (Hossain et al., 2015).

#### 3.2.3 HPLC fingerprints of drying treatment

**FIGURE 1:**
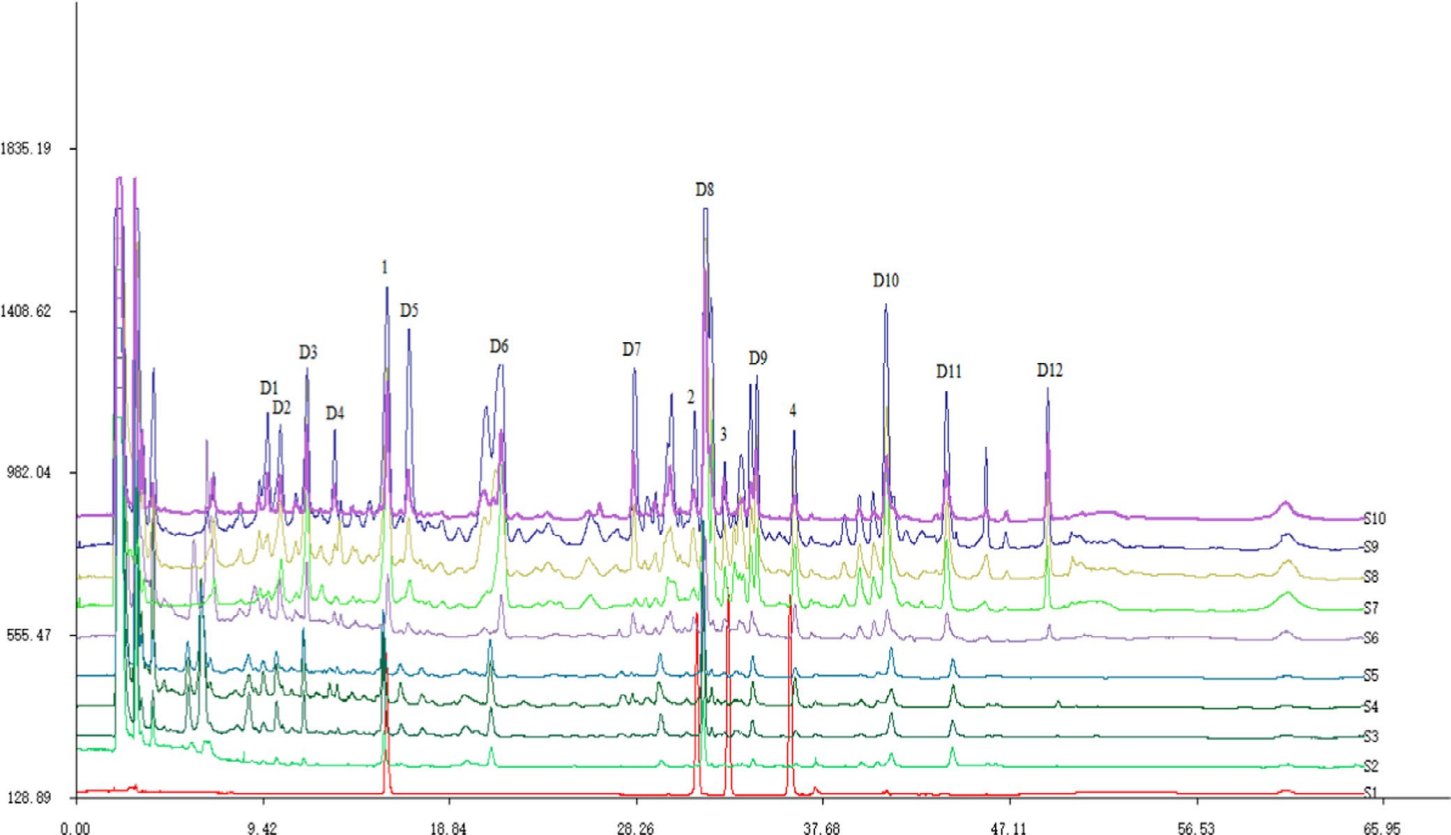
The HPLC fingerprints of drying treatment. Mixed standards (S1), sun drying (S2–S6), and oven drying (S7–S10); peimisine (1), sipeimine (2), peimine (3), and peiminine (4)

Chinese materia medica (CMM) fingerprint technology is an effective method to evaluate the pros and cons, identify the authenticity, distinguish species, and ensure consistency and stability of CMM (Liu et al., 2018). To illustrate the changes of the main active ingredients in the two different postharvest processing methods, we have imported the HPLC‐ELSD chromatogram of the mixed standards (S1), sun drying (S2–S6), and oven drying (S7–S10) samples into the Similarity Evaluation System for Chromatographic Fingerprint of TCM (2004 Editon A) for analysis. The characteristic peaks 1, peak 2, peak 3, and peak 4 were peimisine, sipeimine, peimine, and peiminine, respectively (Figure 1). Other characteristic peaks include D1, D2, D3, D4, D5, D6, D7, D8, D9, D10, D11, and D12, and the contents were calculated based on the peak area of peimisine. Subsequently, the total content of 16 main characteristic peaks was used to evaluate the differences between the two postharvest treatments. We found no significant difference (*p* > .05) in the total content of 16 main characteristic peaks between sun drying and oven drying (Table 4). However, some characteristic compounds such as peimine and peiminine were not detected in the experimental group of H6, P1, and P2. Thus, we inferred that some derivatization and transformation reactions might occur between few compounds during the drying process, but most compounds are relatively stable.

**TABLE 4:** Changes in the contents of 16 main characteristic peaks in bulbs of cultivated *F. cirrhosa* during drying treatment (%, g/g DW)

Sample No.	1	2	3	4	D1	D2	D3	D4	D5	D6	D7	D8	D9	D10	D11	D12	Total
H6	0.01351	0.01713	0.00474	0.00322	0.00143	0.00296	0.00629	_	0.00143	0.00418	0.00341	0.01257	0.00941	0.01132	0.00147	0.00193	0.09500a
P1	0.01406	0.02055	_	_	_	0.00676	0.00493	0.00489	0.00395	0.00369	0.00487	0.01387	0.00679	0.00685	_	0.00098	0.09219a
P2	0.01429	0.01966	_	_	0.00165	_	0.00415	0.00985	0.00267	0.00352	0.00983	0.00956	0.01065	0.00927	0.00256	0.00086	0.09852a
P3	0.01414	0.02068	0.00393	0.00356	0.00243	0.00528	0.00389	0.00698	0.00468	0.00324	0.01240	0.01245	0.00982	0.00289	0.00452	0.00083	0.11172a
P4	0.01372	0.01868	0.00402	0.00331	0.00198	0.00608	0.00368	0.01134	0.00382	0.00229	0.01089	0.01003	0.00683	0.00793	0.00389	0.00075	0.10924a
P5	0.01393	0.01802	0.00443	0.00382	0.00286	0.00789	0.00312	0.00265	0.00416	0.00198	0.01265	0.01212	0.00794	0.00589	0.00476	0.00079	0.10701a
P6	0.01367	0.01793	0.00428	0.00366	0.00223	0.00625	0.00408	0.01164	0.00289	0.00287	0.00876	0.01256	0.01205	0.01368	0.00391	0.00083	0.12129a
P7	0.01378	0.01985	0.00473	0.00391	0.00245	0.00423	0.00351	0.00686	0.00264	0.00165	0.00969	0.01143	0.01097	0.00789	0.00298	0.00075	0.10732a
P8	0.01406	0.01707	0.00448	0.00405	0.00265	0.00505	0.00327	0.00737	0.00379	0.00179	0.01078	0.01367	0.00897	0.00892	0.00428	0.00079	0.11099a
P9	0.01399	0.01821	0.00456	0.00398	0.00296	0.00598	0.00339	0.01162	0.00331	0.00154	0.01263	0.01285	0.01006	0.01102	0.00502	0.00078	0.12190a

Note:

“–” means not determined.

In summary, this study compared the two postharvest processing methods on *F. cirrhosa* bulbs, and the dynamic changes of alkaloids during the drying process were well revealed. Suggested that as a defensive secondary metabolite, the alkaloids in *F. cirrhosa* were related to the plant's reproductive activities and not be easily affected by different processing methods and processing times. Compared with sun drying, oven drying has the advantages of timesaving, continuous operation, and pleasing appearance and color after processing. Therefore, drying in an oven at 60°C for 24 hr can be used as a postharvest processing method for cultivated *F. cirrhosa*.

## 4 CONCLUSIONS

In this study, we dynamically monitored the physicochemical characteristics of *F. cirrhosa* bulbs under different growth stages and different postharvest processing methods. Our results suggested that the best harvest time of the cultivated *F. cirrhosa* was the withering period, and the different processing methods did not show a significant difference in the contents of the active ingredients. Through our research, necessary experimental data were provided to growers and related enterprises for daily production activities and the full utilization and rational development of *F. cirrhosa* resources.

## CONFLICT OF INTEREST

The authors declare that they do not have any conflict of interest.

## ETHICAL APPROVAL

This study does not involve any human or animal testing.

## Data Availability

The data that support the findings of this study are available from the corresponding author upon reasonable request.
